# Intrathyroidal Parathyroid Carcinoma Presenting as Asymptomatic High Normal Serum Calcium and Slightly Elevated Intact Parathyroid Hormone: A Case Report and Review of Literature

**DOI:** 10.4021/wjon311w

**Published:** 2011-06-08

**Authors:** Benjamin Quartey, Craig Shriver, Daniel Russell

**Affiliations:** a National Capital Consortium, National Naval Medical Center, General Surgery Department, 8901 Wisconsin Ave, Bethesda, MD 20889, USA; b National Capital Consortium, Walter Reed Army Medical Center, General Surgery/Surgical Oncology Department, Bldg 2 Rm 5512, 6900 Georgia Ave, Washington, DC 20307, USA; c Department of Pathology, Walter Reed Army Medical Center, Bldg 2, 6900 Georgia Ave, Washington, DC 20307, USA

**Keywords:** Intrathyroid, Parathyroid, Carcinoma, Asymptomatic, Parathyroid hormone

## Abstract

Parathyroid carcinoma is an uncommon endocrine malignancy and the probability of an intrathyroidal location is low. We report a case of intrathyroidal parathyroid carcinoma presenting as asymptomatic high normal serum calcium and slightly elevated intact parathyroid hormone (iPTH) making preoperative suspicion and diagnosis extremely difficult.

## Introduction

Parathyroid carcinoma is a rare entity and accounts for less than 1% of all cases of primary hyperparathyroidism [1-5]. This rare endocrine malignancy was first described by Fritz De Quervain in 1904 in a case of non-functioning metastatic parathyroid carcinoma [6]. Twenty-six years later, Santon and Malegne described the first functional parathyroid carcinoma [7]. These mostly functional tumors are slow growing but progressive; locally invasive but can metastasize to the liver [8]. Their incidence is very low in United States and among the less than 1,000 cases reported in the English literature; only four cases were intrathyroidal in location. This is similar to the incidence of intrathyroidal parathyroid glands overall which ranges from 1.4 - 3.2% at first cervical exploration [9, 10].

Parathyroid carcinoma has equal gender distribution, and is most common in the fourth and fifth decades of life [5, 11]. Most patients present with a palpable neck mass, severe symptoms of primary hyperparathyroidism and hypercalcemia, and a serum intact parathyroid hormone (iPTH) of 3 - 10 times above the upper limit of normal [1, 2, 5]. Knowledge about this rare endocrine malignancy comes from a few case reports and our unique case adds to this limited database. We therefore present an unusual case of intrathyroidal parathyroid carcinoma in an asymptomatic patient with persistent mild hypercalcemia (Calcium 10.4 mg/dl), slightly elevated iPTH (76 pg/ml) and normal neck examination.

## Case Report

**Figure 1:**
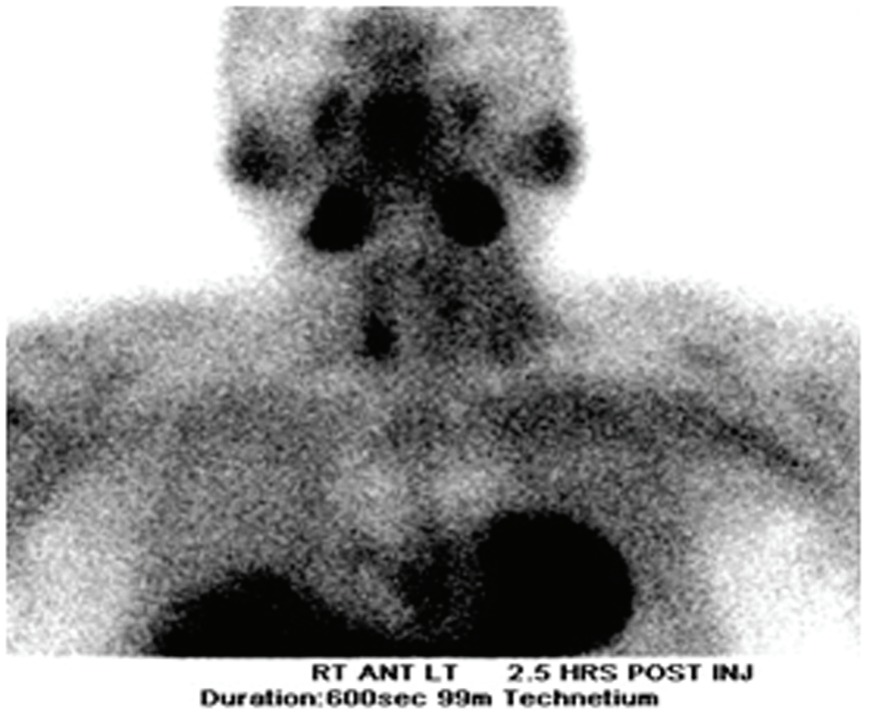
Parathyroid scan

**Figure 2:**
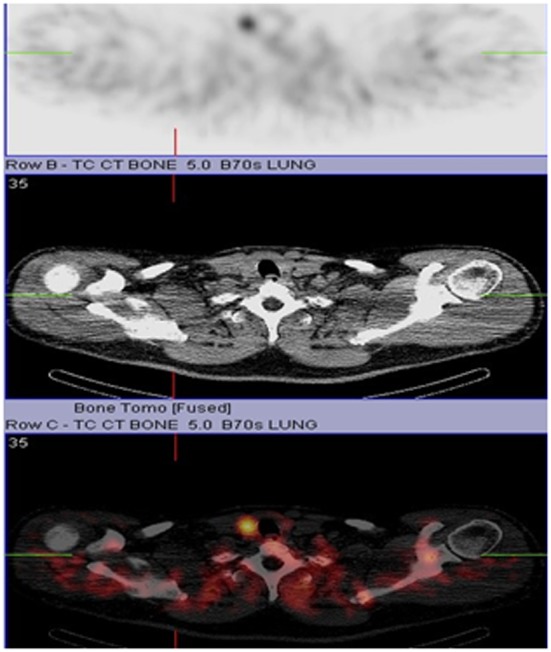
CT/SPECT

**Figure 3:**
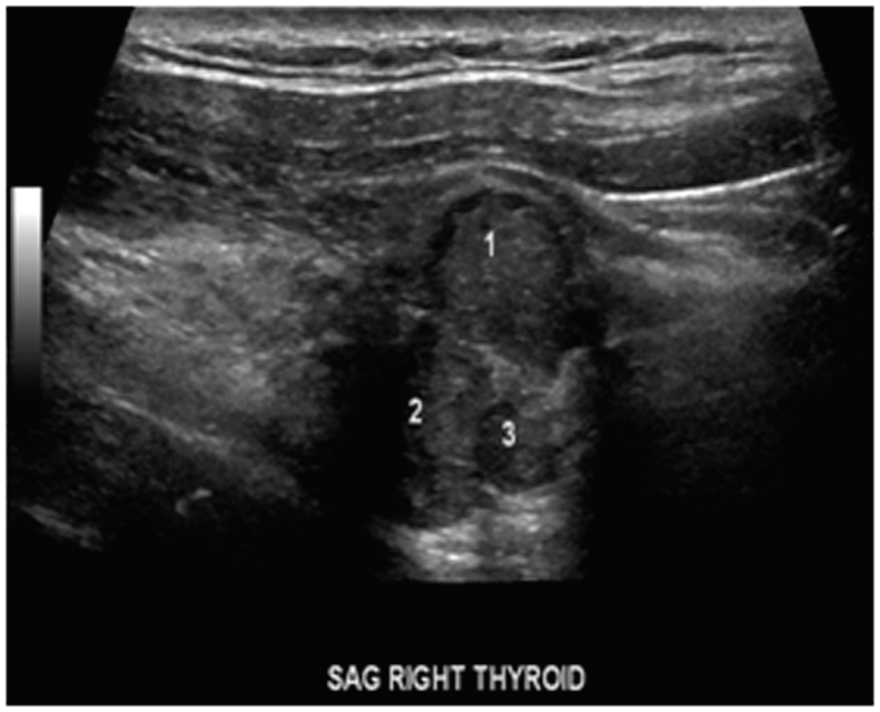
Ultrasound showing parathyroid mass in relation to the two thyroid nodules.

Our patient is a 55-year-old male with history of chronic kidney disease stage III (due to tubulo-interstitial disease, baseline creatinine 1.3 - 1.5), hypothyroidism and hyperlipidemia with diagnosis of primary hyperparathyroidism due to slightly elevated iPTH *(*53 - 67 pg/ml), persistent asymptomatic mild hypercalcemia (10.4 mg/dl), hypercalciuria (379.5 - 484.1 mg/24 hr), hypophosphatemia (2.7 mg/dl), and low 25-OHD (24.6 ng/ml) who was referred to us for parathyroidectomy. There was no personal history of nephrolithiasis, psychiatric complaints or recurrent fractures. Physical exam of the neck was unremarkable by palpation and inspection. Parathyroid imaging scan showed focal uptake in the inferior pole of the right thyroid lobe. DEXA scan was normal and no stones were found on renal ultrasound. Surgical indication was hypercalciuria per NIH criteria [11]. During surgery, the right thyroid lobe was found to have small multiple nodules at the inferior pole corresponding to the area previously localized by preoperative sestimibi with no obvious parathyroid gland. Four gland exploration was performed and three normal appearing parathyroid glands at the usual locations were found except at the right inferior pole. Bilateral neck exploration to include upper mediastinum, the trachea-esophageal groove, the retro-esophageal, and the carotid sheaths was negative. A small area of paratracheal tissue was removed from the right inferior pole and was reported to be a lymph node. Unable to identify a single parathyroid adenoma on full neck exploration, the neck was therefore closed for further localization studies and potential future re-exploration based on results. Preoperative iPTH, postoperative iPTH and serum calcium were 70.12 pg/ml, 71 pg/ml and 10.5 mg/dl respectively. iPTH and serum calcium remained high postoperatively with peak values of 76 pg/ml and 10.7 mg/dl respectively. Repeat parathyroid scan together with single photon emission computed tomography (SPECT) and non-enhanced computer tomography of the upper chest showed increased activity in the mid to inferior portion of the right thyroid lobe at both phases (Fig. 1 and Fig. 2 respectively). Additionally, the SPECT demonstrated increased activity corresponding to a poorly defined hypodensity in the right lobe of the thyroid now suggesting an intrathyroidal parathyroid gland. Thyroid ultrasound showed two hypoechoic nodules or one bi-lobed nodule measuring 1.3 x 1.3 x 1.2 cm (anterior) and 1.1 x 1.2 x 1.2 cm (posterior) which were very close to each other at the inferior right thyroid pole (Fig. 3). Fine needle aspiration with iPTH washings of the anterior thyroid nodule showed iPTH of 236,432 pg/ml (normal non-parathyroid tissue < 30) but cytology was not possible due to insufficient cellularity. Patient was immediately started on cinacalcet 30 mg/day after the above results and the diagnosis was now confirmed as an intrathyroidal parathyroid tumor. He was taken back to the operation room electively and now underwent an uncomplicated right thyroid lobectomy with isthmusectomy. Preoperative and immediate postoperative PTH were 49.19 and 12 pg/ml respectively. Patient had transient decrease in PTH post-operatively and was discharged home with calcium supplement while awaiting PTH to normalize. Patient was seen in clinic postoperative day 13 without complaint, where PTH was 13 pg/ml and serum calcium 9.2 mg/dl. At 3-months follow up, serum was 8.8 mg/dl and doing well. The final pathology specimen revealed intrathyroidal parathyroid carcinoma, with negative margins, and the resection of right paratracheal lymph node at the original operation was negative for any regional nodal metastasis.

### Histopathologic examination

Given the lack of agreed upon morphologic criteria and lack of a uniform diagnostic standard, parathyroid carcinoma (PCA) is a challenging histological diagnosis. The only universally accepted criterion of malignancy is the presence of either local invasion or distant metastasis [12]. There are, however, histopathologic features which help delineate this lesion from parathyroid adenoma, the other main consideration in the differential diagnosis. Generally speaking, adenomas do not display the high nuclear-to-cytoplasmic ratio seen in PCA and usually lack mitotic activity [12-14]. In addition, nucleoli are often regular, small, and inconspicuous, lacking peri-nucleolar halos [12]. Furthermore, the presence of necrosis is unusual in benign parathyroid lesions [12-14]. There are several features in this case that, when taken in total, support a diagnosis of PCA.

**Figure 4:**
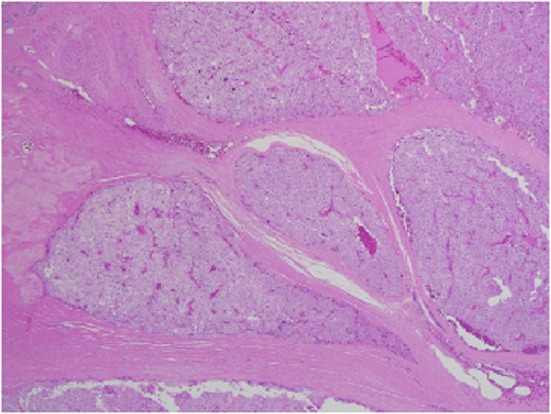
Low power view of parathyroid carcinoma highlighting thick surrounding and intersecting fibrous bands.

**Figure 5:**
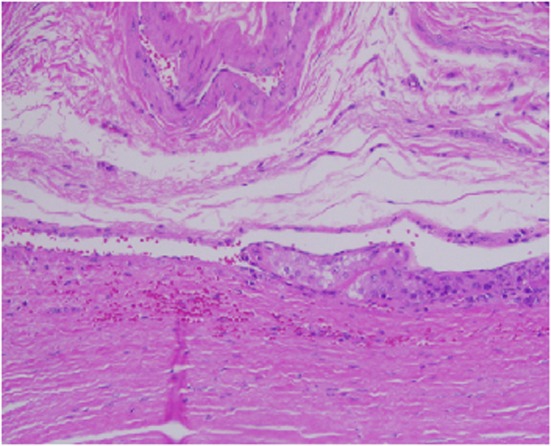
This focus is highly suspicious for invasion of an adjacent vessel by parathyroid carcinoma.

**Figure 6:**
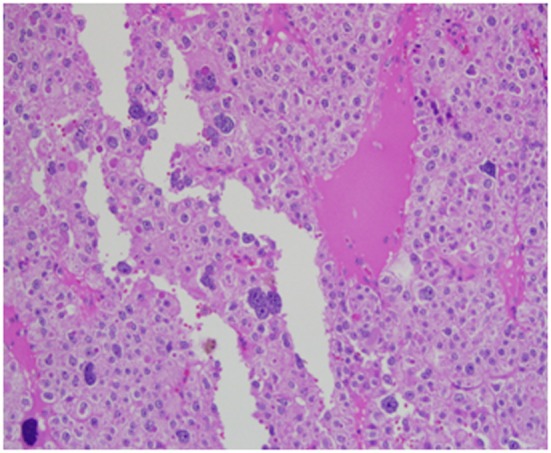
High power view of parathyroid carcinoma highlighting marked nuclear pleomorphism with atypical cells, prominent nucleoli, multinucleation, and abundant droplet filled cytoplasm. The chromatin varies from coarse and clumped in overtly malignant cells with marked pleomorphism to finely stippled (“salt and pepper”) in smaller more typical parathyroid tissue.

**Figure 7:**
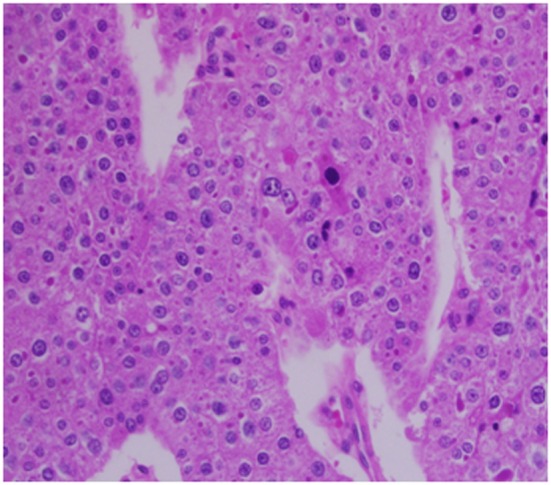
Prominent and irregular eosinophilic nucleoli are seen in this 40 x field with focal perinucleolar halos. The N : C ratios are moderately increased above what is typically seen in parathyroid tissue.

**Figure 8:**
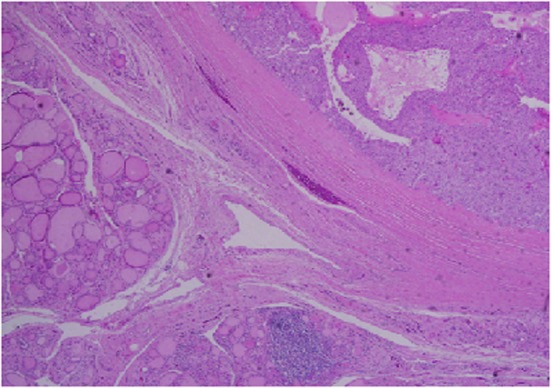
Low power view showing parathyroid carcinoma (upper right) and adjacent thyroid tissue (lower left).

**Figure 9:**
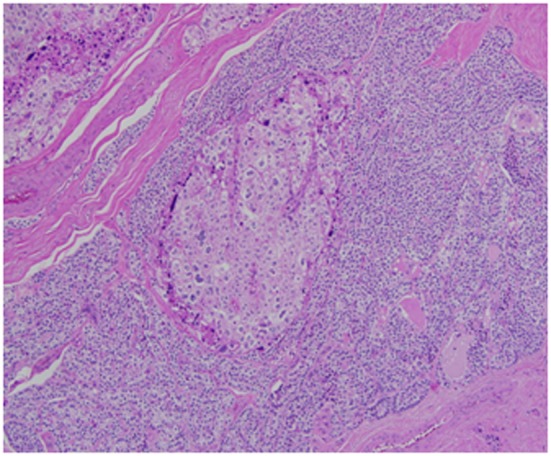
Medium power view of parathyroid carcinoma showing marked nuclear pleomorphic in the background of more typical parathyroid tissue.

Architecturally, the lesion shows a solid to trabecular growth pattern and is associated with hemorrhage and hemosiderin deposition (Fig. 4). In addition, there are foci highly suspicious for lymph-vascular invasion, and the lesion appears to be poorly encapsulated, with islands of tumor separated by thick fibrous bands (Fig. 5). Morphologically, the cytoplasm is clear to amphophilic, with prominent and distinct cell borders. The background cells have monotonous nuclei with neuroendocrine “salt and pepper” chromatin (Fig. 6). The cells of concern range from moderately pleomorphic to wildly atypical and have irregular nuclear contours. In many areas multinucleation is identified. The nucleoli range from inconspicuous to prominent with cherry-red eosinophilia, irregular membranes and peri-nucleolar halos, the latter being a feature almost exclusively seen in PCA [12] (Fig. 7). The sum total of these findings supports a diagnosis of intrathyroidal parathyroid carcinoma (Fig. 8, 9). In this case, the hypercellular parathyroid tissue noted in several of the sections is morphologically distinct from the predominant neoplasm, interpreted as carcinoma (Fig. 9).

## Discussion

Parathyroid cancer is associated with 1 - 3% of all primary hyperparathyroidism cases, however many series had estimated this entity to account for less than 1% [1, 2, 4]. The incidence is about 5% in Italy and Japan [15, 16]. Approximately 30 new cases are reported in the United States yearly according to the National Cancer Data Base [5]. Due to its rarity, preoperative diagnosis is uncommon. The vast majority of patients have functional tumors which can provide clues to a preoperative diagnosis. Most patients present with a palpable neck mass, a very high serum calcium level (average of 14 mg/dl), severe symptoms of primary hyperparathyroidism (kidney stone, weakness, polyuria, polydipsia), and PTH 3 - 10 times above the upper limit of normal [1, 2, 17-19]. The diagnosis is only confirmed by histopathology at resection. Of the less than 1000 cases of parathyroid carcinoma reported in the English literature, only four were intrathyroidal in location. We report the first case in a patient with mild hypercalcemia, slightly elevated PTH, no neck mass and without hypercalcemia symptoms.

In our case, the diagnosis of parathyroid carcinoma was made postoperatively. None of the preoperative objective findings and biochemical profile raised suspicion for parathyroid carcinoma. The lesion was not palpable because of the intrathyroidal location and relatively small size. Hypophosphatemia, which is an uncommon presentation, was seen in this patient. The unique feature of this malignancy is PTH stimulated severe hypercalcemia with complaints of fatigue, malaise, weakness, weight lose, and renal stone. Our patient had none of these symptoms probably due to high normal PTH. Surprisingly, most series report a predilection for inferior parathyroid gland in cases of intrathyroid parathyroid carcinoma as in our case [2].

The standard steps in the management of primary hyperparathyroidism are systematic localization studies followed by minimally invasive parathyroidectomy [20]. A normal parathyroid gland at the location of preoperative studies during surgery dictates four gland and bilateral neck exploration including upper mediastinum, the trachea-esophageal groove, the retroesophageal region, and the carotid sheaths. When this fails to identify the incident gland, then sometimes palpation of thyroid gland for intrathyroid location is recommended. However, a thyroid nodule in the vicinity of the presumed location of parathyroid gland by preoperative studies makes identification for intrathyroid lesion by palpation very difficult if not impossible. Therefore, intrathyroidal parathyroid glands may be missed during imaging or operation as in this case. In this patient, multiple thyroid nodules at the right inferior pole resulted in perceived false positive localization studies. The use of adjunct studies such as SPECT/CT scan and ultrasound provided clues for ectopic location of the parathyroid gland. Although preoperative studies such as ultrasound or CT scan are rarely indicated prior to the initial surgery, we recommend evaluating the thyroid gland with thyroid ultrasound in the management of primary hyperparathyroidism. Imaging is certainly a must when reoperation is being considered in cases of primary hyperparathyroidism after failed exploration because of the anatomic information required that scintigraphy alone is not able to provide [21, 22]. Nevertheless, the utility of these newer technologies remains to be proven with large series. Others proposed that a small palpable mass in the thyroid should be excised via thyrotomy when no pathological gland is found during the initial operation [9]. This would have been very difficult to do at first exploration in our case since the patient had a fibrotic thyroid with multiple nodules at the inferior pole. Therefore, initial right thyroid lobectomy was avoided since the patient was asymptomatic, preoperative chemical values were not impressive, and we were not convinced that the thyroid nodule represented the non-identified parathyroid tumor. We also wanted to re-image the patient with more extensive modalities, to assess for extra-cervical location of parathyroid tissue.

Intrathyroidal parathyroid carcinoma remains a rare entity in hyperparathyroidism. Preoperative biochemical values and nature of symptoms can suggest the diagnosis but this is nonspecific as in our case report. A palpable neck mass which occurs in 50% of patients, severe hypercalcemia (14 mg/dl) and 3 - 10 times the normal values of PTH are potentially indicative of parathyroid carcinoma preoperatively. When no pathological gland is found at the initial surgery, routine bilateral neck exploration is mandatory. If this is negative, thyroid lobectomy with isthmusectomy can be considered if a thyroid nodule is found at the vicinity of the suspected location of the pathological gland.
